# Assessing Animal Welfare and Farm Profitability in Cow-Calf Operations with Stochastic Partial Budgeting

**DOI:** 10.3390/ani11020382

**Published:** 2021-02-03

**Authors:** Haseeb Ahmed, Karin Alvåsen, Charlotte Berg, Helena Hansson, Jan Hultgren, Helena Röcklinsberg, Ulf Emanuelson

**Affiliations:** 1 Department of Clinical Sciences, Swedish University of Agricultural Sciences (SLU), P.O. Box 7054, 750 07 Uppsala, Sweden; karin.alvasen@slu.se (K.A.); ulf.emanuelson@slu.se (U.E.); 2 Department of Animal Environment and Health, Swedish University of Agricultural Sciences (SLU), 532 23 Skara, Sweden; lotta.berg@slu.se (C.B.); jan.hultgren@slu.se (J.H.); 3 Department of Economics, Swedish University of Agricultural Sciences (SLU), 750 07 Uppsala, Sweden; helena.hansson@slu.se; 4 Department of Animal Environment and Health, Swedish University of Agricultural Sciences (SLU), 750 07 Uppsala, Sweden; helena.rocklinsberg@slu.se

**Keywords:** beef production, cow-calf operations, farm animal welfare, profitability, stochastic partial budgeting analysis, suckler beef, Sweden

## Abstract

**Simple Summary:**

The increased consumer demand for high levels of farm animal welfare (FAW) have prompted debates about the costs of achieving higher FAW. Yet, little is known about the economic consequences of improvements in FAW, especially in cow-calf operations. This study contributes to the literature by investigating the relationship between farm profitability and improved FAW measure in terms of increased space allowance in Swedish cow-calf operations. We found that a 0.5 m^2^ increase in space allowance per calf (achieved by a corresponding reduction of herd size) was associated with a 6.9 to 18.7% reduction in farm-level contribution margins in the short term. Our results suggest that FAW improvements can be costly for farms and these costs should be considered while taking policy decisions regarding improvements in FAW standards.

**Abstract:**

The societal demand for good farm animal welfare (FAW) has increased over time. Yet, very little is known about the economic consequences of improvements in FAW in cow–calf operations. This study investigates on-farm economic consequences of improved FAW measures in cow–calf operations. It uses a stochastic partial budgeting approach to examine the relationship between contribution margins and improvements in FAW in terms of increased space allowance for a typical Swedish cow-calf operation, as compared to current practices. In the current practice, a cow should be given at least 5 m^2^ and the calf 2.2 m^2^. We found that a 0.5 m^2^ increase in space allowance per calf (achieved by a corresponding reduction of herd size) was associated with a 6.9 to 18.7% reduction in contribution margins in the short term. Our analysis does not include possible indirect gains like decrease in disease incidence and enhanced non-use or ‘soft’ values associated with increased FAW. However, our analysis indicates that high FAW standards can be costly and careful cost–benefit analysis should be a part of decision-making processes regarding FAW standards. Our results also suggest a need for government support payments and/or the development of market mechanisms to stimulate farmers to continue producing livestock-based foods with high FAW.

## 1. Introduction

High levels of farm animal welfare (FAW) are important for consumer satisfaction and for ensuring ethical standards of animal-based food systems [1,2]. Furthermore, poor FAW is generally considered a negative externality of intensive animal food production. However, farmers in all parts of the world have to deal with several cost and benefit trade-offs when it comes to improving FAW. On the one hand, improvements in FAW may increase profits by ensuring better animal health, higher farm efficiency, and increased value of animals [3,4,5,6,7,8,9]. On the other hand, improving FAW can be costly as it may require changes in housing structure, management routines and/or feeding practices [10,11,12]. However, there is very little understanding of how these trade-offs interact and relate to farm profitability, especially in the cattle meat sector. Evidence-based trade-offs related to FAW are important for understanding cow-calf farmers’ economic incentives for improving FAW, and the extent to which they are likely to undertake improvements with or without policy interventions.

This study uses a typical Swedish cow-calf operation as a case and simulates the relationship between improvement in a selected FAW practice (increase in space allowance) and profitability. Increasing space allowance has been considered important in terms of FAW [13,14]. Informal interviews with extension agents in Sweden prior to undertaking this study also suggested that inadequate space allowance for young animals was a welfare concern at least in some cow-calf operations, making our specific research question important from an economic as well as an animal welfare point of view.

Studies have found positive as well as negative relationships between different FAW indicators and farm profits in the poultry, dairy and pig sectors. Alvåsen et al. (2017) [15] shows that using nurse sows for piglets in Swedish pig production is associated with increased profitability. Gocsik et al. [10] (2016) shows that conventional poultry systems are most cost efficient but rank low on the Animal Welfare Quality index, showcasing a negative relationship between improved FAW and profitability. Stott et al. [16] (2012) shows that improvement in animal welfare score is uncorrelated with farm profitability. However, such studies have not been conducted for the cow-calf operations. This paper aims to estimate the economic consequences of improved FAW in these types of farms. In doing so, the paper adds to the debate on FAW standards and their impacts on farm profitability, which is of economic and public policy relevance globally. In addition, by estimating the relationship between changes in resource-based FAW standards and contribution margins and discussing broader economic implications of such standards, the study contributes more generally to the literature on the economic implications of food standards, which are becoming more common as the societal push for environmentally and socially conscious food systems gain momentum.

## 2. Materials and Methods

### 2.1. Background

A typical Swedish cow-calf operation usually consists of about 20 suckler cows and about 20 calves that are reared for sale to fattening [17]. The calves of a typical operation are usually born in the spring and are reared for about 6 months together with their mothers. It is not a legal requirement to rear calves on pasture until they are 6 months old. However, cows are required to spend time on pasture. Calves below 6 months of age could thus be raised indoors as well as grazed outdoors with the dams. Grazing is an important and often practiced management routine in these types of farms. In different parts of Sweden, the minimum legal requirement for grazing time varied between 2 and 4 months [18].

In terms of indoor space allowance, the minimum legal requirement for a suckler cow on its own is 5 m^2^ [18]. The legal requirement of indoor space allowance for the calf alone is 2.2 m^2^ per calf in group housing, given that the live weight of the calf is less than 150 kg [18]. We modelled a new (hypothetical) space allowance policy, where calves had an additional 0.5 m^2^/calf (a 25% increase) of space in all weight categories less than 150 kg.

### 2.2. Scenarios

To assess the net change in contribution margin associated with changes in space allowance policy, we assumed that the policy change was expected and implemented after giving enough time for the farmers to adjust their production cycles. This implied that farmers did not have to sell calves suddenly but had the time to plan and reduce herd size by reducing pregnancies and/or selling cows. Furthermore, we assumed that the farm was operating at full capacity and that any increase in mandatory space allowance for calves would decrease the calf or cow herd size, at least in the short term. We built two scenarios that corresponded to the likely short-term response of farmers when faced with a change in space allowance policy.

In Scenario 1, we assumed that farmers adjusted their production cycles by reducing pregnancies in their cows. We also assumed that the barn had 44 m^2^ of space for 20 calves (complying with Swedish FAW legislation), and that the increase of 0.5 m^2^/animal was accomplished by reducing calf herd size by four calves. This scenario was further divided into two sub-scenarios, where calves were raised indoors in Scenario 1a, while Scenario 1b took 90 days of grazing into account (an average of legally required periods in different regions of Sweden).

In Scenario 2, we assumed that the farmers responded to a change in required space allowance by removing a cow and a subsequent calf from the farm. Indeed, if a farmer cannot keep a calf he/she has little use for the ‘idle’ dam. Reducing a cow and calf from the herd would create 7.7 m^2^ (5 m^2^ for the mother cow and 2.7 m^2^ for the calf, under the new policy) of free space. In this scenario, the farm would still have to reduce calf herd size by an additional unit, as only two calves would fit into the extra 7.7 m^2^. Hence, as far as the calf herd was concerned, the policy would lead to a reduction of herd size by two. This scenario was also divided into two sub-scenarios, where Scenario 2a meant that calves were raised indoors, while Scenario 2b took grazing time into account in the same way as Scenario 1b.

### 2.3. Data and Calculations

Simulation models were constructed for all four scenarios in Microsoft Excel (Microsoft, Redmond, WA, USA). The basic model was constructed using costs and revenue data from a database of accounting data for several types of farms ([19]) and biological parameters from the literature [13,20,21,22]. The model estimated the net benefits and costs of an increased space allowance within a partial budgeting framework. Table 1 provides information about the data (obtained from [19]) and distributions of biological parameters (obtained from the literature).

Benefits and costs were estimated as:

Net change = (Increased Income + Reduced Cost) − (Increased Cost + Reduced Income)(1)

where

Reduced Income = Reduction in Calf Herd Size × Per Calf Contribution Margin at Baseline(2)

Increased Income = Number of Calves on the Farm × Increase in DWG × Number of Production Days Spent Indoors × Ratio of Live to Carcass Weight × Price per Kg of Beef(3)

Increased Cost = Number of Calves on the Farm × Increase in Silage Intake × Price of Silage + Number of Calves on the Farm × Increase in Grain Intake × Price of Grain(4)

Reduced cost was not applicable to our model. Indeed, this is a restrictive assumption of our model. Increased space allowance and decreased herd size may be associated with reductions in disease incidence as well as labor requirements. However, we did not find good estimates of the relationship between disease incidence and space allowance and were unable to capture these reductions in costs. Similarly, we did not have information on how labor requirements change with herd size and therefore we did not include that in the model. Furthermore, decrease in cow herd size in Scenarios 2a and b would be accompanied with decrease in feeding costs. These costs are considered in one of our robustness checks (see Results section for more details).

Reduction in income (Equation (2)) stems from the farmer’s inability to maintain the baseline calf herd size. The added income was calculated by multiplying additional weight gain per calf per day, the number of production days, price per kg and number of calves on the farm, assuming that output price per kg was not affected under the new policy regime (as illustrated in Equation (3)). The total number of production days spent indoors, for cases where the calves were reared indoors (Scenarios 1a and 2a), was 180. However, the number of production days for calves reared on pasture was 90 (the assumed grazing period). It was further assumed that expected live daily weight gain (DWG) would be 32 g higher under the new policy [20] in these scenarios. In contrast, no difference in growth was expected in the baseline vs. policy change scenario during the grazing period, provided that everything else was equal.

Given the stochastic nature of growth rate and lack of information about the actual distribution of this parameter, it was modeled using a triangular distribution with 22 g/day as lower bound, 32 g as the mode and 33.7 g/day as upper bound. The lower bound and mode were chosen from the DWG reported in [20] and the upper bound was chosen by the estimates reported in [13]. To estimate the corresponding gain in carcass weight, the distribution of live DWG was multiplied by 63% [21].

The added cost of increased feed intake was calculated by multiplying the increase in silage and grain intake per calf with corresponding prices of silage and grain (Equation (4)). We assumed that increased space allowance would increase silage and grain intake per calf by 2.5% per day, modeled stochastically with a triangular distribution with 1.25% as the lower bound, 2.5% as mode, and 3.75% as the upper bound [22]. Parameter estimates used for daily weight gain and feed intake were obtained from studies done in Finland and Denmark, where the breeds of animals, production systems and practices are likely to be similar to Sweden. Since we had credible information on mode and upper and lower bounds, but not the actual distribution of this variable, a triangular distribution was considered most appropriate to use [11,15].

Price data were also collected from [19]. The price of silage was modeled using a triangular distribution with 0.94 Swedish Kronor (SEK)/kg as lower bound, 1.25 SEK/kg as mode and 1.41 SEK/kg as upper bound. Similarly, the price of grain was modeled using a triangular distribution with 1.35 SEK/kg as lower bound, 1.38 SEK/kg as mode and 1.41 SEK/kg as upper bound. Output prices of meat were also modeled stochastically using a triangular distribution with 32.25 SEK/kg as lower bound, 33.91 SEK/kg as mode and 35 SEK/kg as upper bound (Table 1).

A sensitivity analysis of the impact of the stochastic factors on outcome values for all four scenarios was performed using the @Risk (Palisade, Ithaca, NY, USA) add-in in Microsoft Excel. A simulation of 5000 iterations was run and a tornado plot with regression coefficients was created. The @Risk program runs a multiple regression analysis using one value per iteration with the output of interest as the dependent variable and the simulated values of the stochastic variables as independent variables. In our model, the change in contribution margin is the dependent variable and stochastic variables such as (input and output) prices, feed intake and daily weight gain are independent variables. The length of the bars in the Tornado plot represent the importance of variables that drive the changes in net contribution margin.

## 3. Results

Scenario 1a: The results from the partial budget model for cases where the farmer responds with reducing only the calf herd size and calves are reared indoors are provided in Table 2. With a 0.5 m^2^/calf increase in space allowance, the income for the farm increased by 2024 (SD = 287.8) SEK per year due to the increase in DWG (from Equation (3)). However, this increase in income was offset by an increase in costs due to increased feed (silage and grains) intake and reduction of income due to decreased herd size. The reduction in income due to decreased herd size was 10,076 [= 2519 × 4] SEK (Equation (2)). The increase in silage and grain costs was 639 (91.1) and 35 (3.9) SEK, respectively (Equation (4)). The total decrease in benefit (increased costs + reduced income) from the policy change was 10,750 (92.0) SEK per year in the short term.

**Figure 1:**
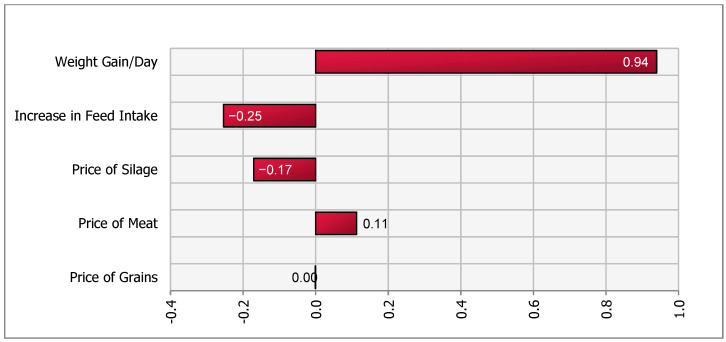
Tornado plot of regression coefficients for determinants of a change in net income under increased space allowance (from 2.2 to 2.7 m^2^/calf)—Scenario 1a.

Therefore, a 0.5 m^2^/calf increase in mandatory space allowance was associated with a net change in contribution margin of −8726 (303.4) SEK per year as compared to the baseline (Equation (1)). Hence, the contribution margin after the policy change in this scenario was 41,654 SEK. This represented a reduction in herd-level contribution margin from 50,380 [20 × 2519] SEK to 41,654 SEK. Thus, a reduction in a herd-level contribution margin of 17.3% $\left[\left(1-\frac{41,654}{50,380}\right)\times 100\right]$ as compared to the baseline. Figure 1 suggested that DWG was the most important variable driving the contribution margin. The decrease in herd size, assumed to be deterministic, was also an important variable that reduced revenue and negatively affected the herd-level contribution margin in the short term. Our simulation yielded a range of net change in contribution margins between −7186 and −9200 SEK.

Scenario 1b*:* The results from the partial budget model for cases where farmer responds with reducing only the calf herd size and calves spend 90 days grazing are provided in Table 3. With a 0.5 m^2^/calf increase in space allowance, the income for the farm increased by 1012 (SD = 143.9) SEK per year due to the increase in DWG (from Equation (3)). However, this increase in income was offset by an increase in costs due to increased feed (silage and grains) intake and reduction of income due to decreased herd size. The reduction in income due to decreased herd size was 10,076 [= 2519 × 4] SEK (Equation (2)). The increase in silage and grain costs was 320 (69.1) and 17 (3.5) SEK, respectively (Equation (4)). The total decrease in benefit (increased costs + reduced income) from the policy change was 10,413 (72.4) SEK per year in the short term.

**Figure 2:**
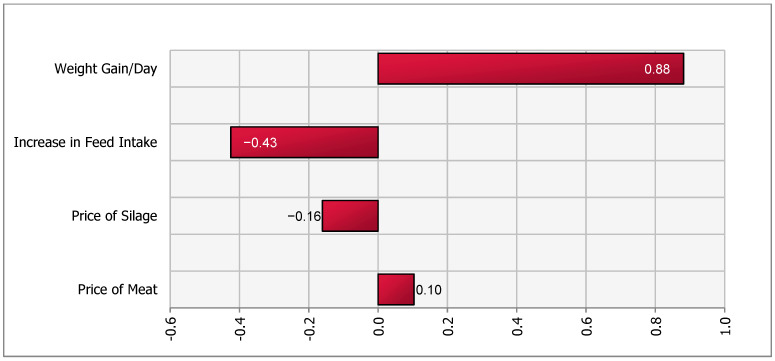
Tornado plot of regression coefficients for determinants of a change in net income under increased space allowance (from 2.2 to 2.7 m^2^/calf)—Scenario 1b.

Therefore, a 0.5 m^2^/calf increase in mandatory space allowance was associated with a net change in a contribution margin of −9401 (164.8) SEK per year (Equation (1)). Hence the contribution margin after the policy change in this scenario was 40,979 SEK. This represented a reduction in herd-level contribution margin from 50,380 [20 × 2519] SEK to 40,979 SEK. Thus, a reduction in a herd-level contribution margin of 18.66% $\left[\left(1-\frac{40,979}{50,380}\right)\times 100\right]$. Figure 2 suggested that DWG was the most important variable driving the contribution margin. The decrease in herd size, assumed to be deterministic, was an important variable that reduced revenue and negatively affected the herd-level contribution margin in the short term. Our simulation yielded a range of net change in contribution margins between −8918 and −9894 SEK.

Scenario 2a: Results from the partial budget model for cases where farmer responds to the policy change by reducing the cow as well as calf herd size (and calves are reared indoors) are provided in Table 4. With a 0.5 m^2^/calf increase in space allowance, the income for the farm increased by 2277 (323.7) SEK per year due to the increase in DWG associated with increased space allowance (using Equation (3)). However, this increase in income was offset by an increase in costs due to increased feed (silage and grains) intake and reduction of income due to decreased calf herd size. The reduction in income due to decreased herd size was 5038 [= 2519 × 2] SEK (Equation (2)). The increase in silage and grain costs was 719 (101.3) and 39 (4.5) SEK, respectively (Equation (4)). The decrease in benefits (increased costs + reduced income) was 5796 (105.5) SEK per year, in the short term.

Therefore, a 0.5 m^2^/calf increase in mandatory space allowance was associated with a net change in the contribution margin of −3520 (339.9) SEK per year. Hence, the contribution margin after the policy change in this scenario was 46,860 SEK. This was a reduction in herd-level contribution margin from 50,380 (20 × 2519) SEK to 46,855 SEK. A reduction in a herd-level contribution margin of 6.9 % $\left[\left(1-\frac{46,860}{50,380}\right)\times 100\right]$. Additionally, there may be some revenue from selling the cow that was supposed to produce a calf in the baseline scenario. However, that revenue would be temporary in nature and our contribution margin calculations reflect a fairly short-term steady-state. Furthermore, we are mainly interested in income from selling calves, which is the main product of these kinds of operations. Therefore, the revenue from selling that cow is not taken into account in our calculations. There may be some cost savings from not feeding the removed cow. We used an average of 1200 SEK per year [19] as cost savings in one of our robustness checks. While the reduction in contribution margin did go down, the relationship between increased space allowance and contribution margin still remained negative.

**Figure 3:**
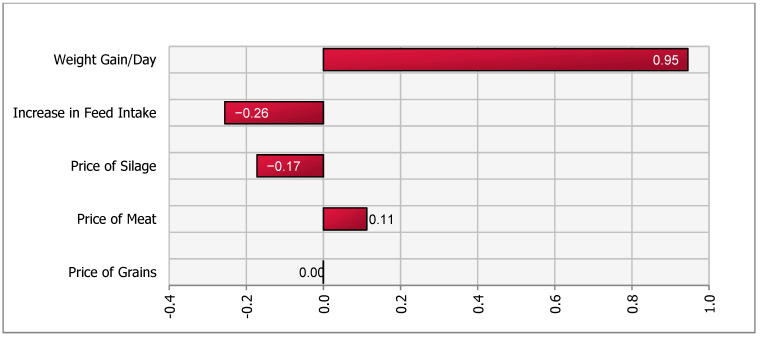
Tornado plot of regression coefficients for determinants of a change in net income under increased space allowance (from 2.2 to 2.7 m^2^/calf)—Scenario 2a.

Sensitivity analysis suggested that DWG was the most important variable driving the contribution margin (Figure 3). The decrease in herd size, assumed to be deterministic, was an important variable that reduced revenue and negatively affected the herd-level contribution margin in the short term. Our simulation yielded a range of net change in contribution margins between −2538 and −4467 SEK.

Scenario 2b: Results from the partial budget model for cases where farmer responds to the policy change by reducing the cow as well as calf herd size (and calves are reared on pasture for 90 days) are provided in Table 5. With a 0.5 m^2^/calf increase in space allowance, the income for the farm increased by 1138 (161.1) SEK per year due to the increase in DWG associated with increased space allowance (using Equation (3)). However, this increase in income was offset by an increase in costs due to increased feed (silage and grains) intake and reduction of income due to decreased calf herd size. The reduction in income due to decreased herd size was 5038 [= 2519 × 2] SEK (Equation (2)). The increase in silage and grain costs were 360 (79.2) and 20 (3.9) SEK, respectively (Equation (4)). The decrease in benefits (increased costs + reduced income) was 5417 (82.9) SEK per year in the short term.

Therefore, a 0.5 m^2^/calf increase in mandatory space allowance was associated with a net change in the contribution margin of −4279 (181.7) SEK per year. Hence, the contribution margin after the policy change in this scenario was 46,101 SEK. This was a reduction in herd-level contribution margin from 50,380 (20 × 2519) SEK to 46,101 SEK. A reduction in a herd-level contribution margin of 8.49% $\left[\left(1-\frac{46,101}{50,380}\right)\times 100\right]$.

**Figure 4:**
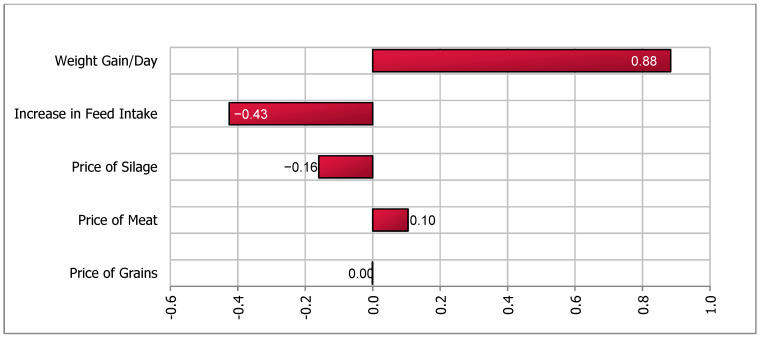
Tornado plot of regression coefficients for determinants of a change in net income under increased space allowance (from 2.2 to 2.7 m^2^/calf)—Scenario 2b.

Sensitivity analysis (Figure 4) suggested that DWG was the most important variable driving the contribution margin. The decrease in herd size, assumed to be deterministic, was an important variable that reduced revenue and negatively affected the herd-level contribution margin in the short term. Our simulation yielded a range of net change in contribution margins between −3694 and −4825 SEK.

## 4. Discussion and Policy Implications

An increase in space allowance was associated with a reduction in the contribution margin of the farm by 6.9–18.7%, depending on the scenario, in the short term. Scenarios 2a and b, where the farmer reduces the cow and calf herd size, seems to minimize the losses from the policy change and may be preferred by the farmer. However, we acknowledge that these scenarios require farmers to keep ‘idle’ cows, which might be costly.

The reduction in contribution margin in all four scenarios was mainly attributed to the lower number of calves in the herd. We assumed that cow-calf operations were unable to increase the size of the barn, were operating at maximum capacity in terms of herd size, and therefore had to reduce calf or cow-calf herd size in order to increase space per calf. Sensitivity analysis (Figure 1, Figure 2, Figure 3 and Figure 4), for all scenarios, suggested that DWG associated with an increase in space allowance was the main stochastic determinant of change in contribution margins. The stochastic approach used in our partial budgeting accounted for parameter variation and generated results with a distribution, representing uncertainty in results. Additionally, stochastic modeling allowed us to investigate the most important associations between stochastic parameters (prices, feed intake or DWG) and changes in contribution margin (tornado plots of regression coefficients shown in Figure 1, Figure 2, Figure 3 and Figure 4).

A negative relationship between improved FAW and contribution margins has several economic and societal implications. First, in the absence of governmental or private financial incentives for improved FAW, and assuming constant output prices, costs associated with more stringent FAW legislation can drive cost-inefficient farms out of business and leave fewer but likely larger farms to capture the available market share [23]. This may be considered unproblematic if we only consider the private benefits of farming. However, the presence of cattle farms across Sweden provides social and environmental amenities in terms of agrotourism, biodiversity and maintaining and developing the countryside. Therefore, a loss of farms due to the cost of FAW legislation could adversely affect some of the advantages of cow-calf farms.

Second, costs associated with more stringent FAW standards can make Swedish beef less competitive compared to imported beef unless farmers can affect demand and realize higher output prices by better by communicating the value added to products [12]. Imported beef may not be produced with similar strict FAW standards and its consumption may stimulate and even force Swedish farmers to become price competitive. This could induce cost-cutting measures and take farmers’ attention away from providing higher FAW and producing better value-added products. Hence, an overall (and probably unintended) impact of a costly FAW legislation could be that farmers supply less-than socially desired FAW.

Farmers could potentially market these products with higher FAW as differentiated products. However, in our models, the average price per kg would have to more than double for the farmer to be compensated for the loss in short-term contribution margins. Moreover, despite a stronger will to pay for FAW attributes in food production [6,7,8], price premiums in the food value chain may not translate into higher output prices to farmers [9], and farmers’ direct access to consumers may not always exist. Given these results and their implications for FAW, support payments for FAW can become an important tool to address the negative externalities associated with poor or less-than socially optimal provision of FAW. In addition, such support payments may stimulate farmers to continue improving FAW in the face of rising costs, especially when market solutions alone cannot mitigate poor FAW and ensure sufficient provision of good FAW. Indeed, there are now initiatives in some countries that provide monetary support for maintaining higher FAW, e.g., ‘Initiative Tierwohl’ in Germany [24].

In Sweden, there are currently no official payments for specific animal welfare practices, although in some parts of the country beef from cattle grazing on natural pastures is labeled and sold at a premium price. There is, furthermore, an organic labeling scheme that allows farmers to get higher prices for their products by complying with higher FAW, environmental and social standards. But it is uncertain to what degree consumers pay specifically for the FAW attributes of such labels. Furthermore, even if the prices for animal welfare attributes increase, only part of that increased premium will reach the farmers because of the way food value chains are currently structured [9]. Therefore, there is a need for more research regarding the consumers’ willingness to pay for separate animal welfare labels and farmers’ willingness to invest in specific FAW attributes, and the overall impact of such attributes on-farm profitability. From a public policy perspective, there may be a need for a more diversified set of payments that can stimulate improvements in FAW more directly.

There is limited work done in the domain of FAW in suckler herds, which limits the input variables used in the analysis. For example, the impact of increased space allowance on calf health in cow-calf settings was an underreported topic in the literature. Consequently, we have not been able to capture the mortality and morbidity aspects (e.g., the potential decrease in risk of respiratory disease due to increased air space) related to an increase in space allowance given limited data, especially for cow-calf herds. Similarly, indirect values through e.g. decreased disease incidence and enhanced non-use or ‘soft’ values were not a part of the analysis. In that sense, our model perhaps overestimated the negative relationship between space allowance and contribution margins and/or left out other benefits of improved FAW. There are of course also other potential FAW improvements, which may not have a similar effect on contribution margins. In addition, long-term consequences and the relationship between FAW, meat quality and demand, and farmers’ perceptions and motivation to improve FAW are important subjects for future research.

## 5. Conclusions

This study investigated the economic consequences of measures to improve farm-animal welfare in cow-calf operations. We found that a 0.5 m^2^ increase in space allowance per calf (achieved by a corresponding reduction of herd size) was associated with a 6.9 to 18.7% reduction in contribution margins in the short term (when only pecuniary and direct effects are considered). Our work emphasizes the need for careful cost–benefit analysis prior to making any policy decisions on FAW standards. Future work should focus on including indirect gains of FAW in terms of enhanced non-use values and reduction of disease incidence to improve upon the precision of existing cost–benefit estimates.

## Figures and Tables

**Table 1:** Overview of the variables used in partial budget models for increased space allowance (from 2.2 to 2.7 m^2^/calf).

Variable	Data	Source	Type	Distribution
Decrease in calf herd size (Scenarios 1a and b)	4 heads	-	Deterministic	-
Decrease in calf herd size (Scenarios 2a and b)	2 heads	-	Deterministic	-
Production days	180 days	Agriwise (2018) [19]	Deterministic	-
Grazing period	90 days	-	Deterministic	-
Contribution margin per calf in base scenario	2519 SEK	Agriwise (2018) [19]	Deterministic	-
Increase in daily weight gain at increased space allowance	32 g/day	Andersen et al. (1997) [20] and Herva et al. (2009) [13]	Stochastic	Triangular (22, 32, 33.7)
Increase in feed intake	2.5 %/day	Ingvartsen and Andersen (2009) [22]	Stochastic	Triangular (1.25, 2.5, 3.75)
Price of meat	33.91 SEK/kg	Agriwise (2018) [19]	Stochastic	Triangular (32.25, 33.91, 35)
Price of silage	1.25 SEK/kg	Agriwise (2018) [19]	Stochastic	Triangular (0.94, 1.25, 1.41)
Price of grains	1.38 SEK/kg	Agriwise (2018) [19]	Stochastic	Triangular (1.35, 1.38, 1.41)

**Table 2:** Effects ^a^ of an increase in space allowance (from 2.2 to 2.7 m^2^/calf)—Scenario 1a.

Category	Item	SEK
Added benefit due to change	Benefit due to weight gain	2024 (± 287.8)
Reduced costs due to change	None	-
Increase in contribution margin = Added benefit + Reduced cost		2024 (± 287.8)
Added costs due to change	Costs due to the increase in silage intake	−639 (± 91.1)
	Costs due to the increase in grain intake	−35 (± 3.9)
Reduced benefit due to change	Income forgone due to reduction in calves	−10,076
Decrease in contribution margin = Added cost + Reduced benefit		−10,750 (± 92.0)
Net Change in Contribution Margin		−8726 (± 303.4)

^a^ The values presented are means ±standard deviations for stochastic components in parenthesis.

**Table 3:** Effects ^a^ of an increase in space allowance (from 2.2 to 2.7 m^2^/calf)—Scenario 1b.

Category	Item	SEK
Added benefit due to change	Benefit due to weight gain	1012 (± 143.9)
Reduced costs due to change	None	-
Increase in contribution margin = Added benefit + Reduced cost		1012 (± 143.9)
Added costs due to change	Costs due to the increase in silage intake	−320 (± 69.1)
	Costs due to the increase in grain intake	−17 (± 3.5)
Reduced benefit due to change	Income forgone due to reduction in calves	−10,076
Decrease in contribution margin = Added cost + Reduced benefit		−10,413 (± 71.3)
Net Change in Contribution Margin		−9401 (± 164.8)

^a^ The values presented are means ±standard deviations for stochastic components in parenthesis.

**Table 4:** Effects ^a^ of an increase in space allowance (from 2.2 to 2.7 m^2^/calf)—Scenario 2a.

Category	Item	SEK
Added benefit due to change	Benefit due to weight gain	2277 (± 323.7)
Reduced costs due to change	None	-
Increase in contribution margin = Added benefit + Reduced cost		2277 (± 323.7)
Added costs due to change	Costs due to the increase in silage intake	−719 (± 101.3)
	Costs due to the increase in grain intake	−39 (± 4.5)
Reduced benefit due to change	Income forgone due to reduction in calves	−5038
Decrease in contribution margin = Added cost + Reduced benefit		−5796 (± 104.9)
Net Change in Contribution Margin		−3520 (± 339.9)

^a^ The values presented are means ±standard deviations for stochastic components in parenthesis.

**Table 5:** Effects ^a^ of an increase in space allowance (from 2.2 to 2.7 m^2^/calf)—Scenario 2b.

Category	Item	SEK
Added benefit due to change	Benefit due to weight gain	1138 (± 161.1)
Reduced costs due to change	None	-
Increase in contribution margin = Added benefit + Reduced cost		1138 (± 161.1)
Added costs due to change	Costs due to the increase in silage intake	−360 (± 79.2)
	Costs due to the increase in grain intake	−20 (± 3.9)
Reduced benefit due to change	Income forgone due to reduction in calves	−5038
Decrease in contribution margin = Added cost + Reduced benefit		−5417 (± 82.9)
Net Change in Contribution Margin		−4279 (± 181.7)

^a^ The values presented are means ±standard deviations for stochastic components in parenthesis.

## Data Availability

Data presented in this study is available on request from the corresponding author.
